# Inhibition of APP gamma-secretase restores Sonic Hedgehog signaling and neurogenesis in the Ts65Dn mouse model of Down syndrome

**DOI:** 10.1016/j.nbd.2015.08.001

**Published:** 2015-10

**Authors:** Andrea Giacomini, Fiorenza Stagni, Stefania Trazzi, Sandra Guidi, Marco Emili, Elizabeth Brigham, Elisabetta Ciani, Renata Bartesaghi

**Affiliations:** a Department of Biomedical and Neuromotor Sciences, University of Bologna, Bologna, Italy; b Elan Pharmaceuticals, South San Francisco, CA, USA

**Keywords:** Down syndrome, Neonatal pharmacotherapy, APP, AICD, γ-Secretase

## Abstract

Neurogenesis impairment starting from early developmental stages is a key determinant of intellectual disability in Down syndrome (DS). Previous evidence provided a causal relationship between neurogenesis impairment and malfunctioning of the mitogenic Sonic Hedgehog (Shh) pathway. In particular, excessive levels of AICD (amyloid precursor protein intracellular domain), a cleavage product of the trisomic gene APP (amyloid precursor protein) up-regulate transcription of Ptch1 (Patched1), the Shh receptor that keeps the pathway repressed. Since AICD results from APP cleavage by γ-secretase, the goal of the current study was to establish whether treatment with a γ-secretase inhibitor normalizes AICD levels and restores neurogenesis in trisomic neural precursor cells. We found that treatment with a selective γ-secretase inhibitor (ELND006; ELN) restores proliferation in neurospheres derived from the subventricular zone (SVZ) of the Ts65Dn mouse model of DS. This effect was accompanied by reduction of AICD and Ptch1 levels and was prevented by inhibition of the Shh pathway with cyclopamine. Treatment of Ts65Dn mice with ELN in the postnatal period P3–P15 restored neurogenesis in the SVZ and hippocampus, hippocampal granule cell number and synapse development, indicating a positive impact of treatment on brain development. In addition, in the hippocampus of treated Ts65Dn mice there was a reduction in the expression levels of various genes that are transcriptionally regulated by AICD, including APP, its origin substrate. Inhibitors of γ-secretase are currently envisaged as tools for the cure of Alzheimer's disease because they lower βamyloid levels. Current results provide novel evidence that γ-secretase inhibitors may represent a strategy for the rescue of neurogenesis defects in DS.

## Highlights

• Derangement of the mitogenic Shh pathway reduces neurogenesis in Down syndrome (DS).
• APP triplication causes excessive formation of its cleavage products AICD.
• AICD causes excessive transcription of Ptch1, the repressor of the Shh pathway.
• ELND006, a gamma secretase inhibitor, reduces AICD levels and Ptch1 expression.
• Treatment with ELND006 restores neurogenesis in the Ts65Dn mouse model of DS.

## 1 Introduction

Down syndrome (DS) is a genetic condition caused by the triplication of human chromosome 21. Intellectual disability is the unavoidable hallmark of DS, with a heavy impact on public health. In DS patients the average IQ score is around 50 (range: 30–70) (Creau, 2012, Gardiner, 2015) and in adults with DS it may also be influenced by the increased risk of early onset dementia of the Alzheimer's type (Creau, 2012, Gardiner, 2015, Roizen and Patterson, 2003).

Generalized neurogenesis impairment during critical developmental stages and impaired dendritic maturation are the major determinants of intellectual disability in individuals with DS. Evidence obtained in mouse models of DS with triplication of different sets of genes or individual genes shows that these models may exhibit a similar brain phenotype, suggesting that different genes may contribute to the same brain phenotypic feature. Consistently with this idea, therapeutic approaches targeted to trisomy-linked alterations of different pathways can improve/rescue the same brain phenotype (e.g. neurogenesis alterations).

Accumulating evidence in the Ts65Dn mouse model of DS suggests that alteration of the Sonic Hedgehog (Shh) pathway may be one important factor involved in neurogenesis impairment in DS (Roper et al., 2006, Trazzi et al., 2011, Trazzi et al., 2013). In particular, defective functioning of the Shh pathway appears to cause reduced proliferation of neural precursor cells (NPCs) of the cerebellum (Baxter et al., 2000, Roper et al., 2006), the subventricular zone (SVZ) of the lateral ventricle (Trazzi et al., 2011, Trazzi et al., 2013) and the subgranular zone (SGZ) of the hippocampal dentate gyrus (Trazzi et al., 2011). Up-regulation of the Shh pathway normalizes some developmental defects in Ts65Dn mice (Dutka et al., 2015). Regarding the causes of Shh signaling impairment in DS, recent data suggest that the triplicated gene APP (amyloid precursor protein), a gene that is important for cell cycle progression and neuron migration (Nalivaeva and Turner, 2013), may be a key candidate underlying trisomy-linked alteration of Shh signaling (Trazzi et al., 2013).

APP undergoes complex proteolytic processing, giving rise to several fragments. Cleavage of APP by α- and β-secretases gives origin to the carbossiterminal fragments (CTFs) αCTF and βCTF, respectively. Cleavage of αCTF by the enzyme γ-secretase gives origin to the amyloid precursor protein intracellular domain (AICD) and p3, and cleavage of βCTF gives origin to βamyloid (Aβ) and AICD. We previously found that excessive AICD levels in trisomic NPCs caused over-expression of Patched 1 (Ptch1), an Shh receptor that keeps the Shh pathway in a repressed state (Trazzi et al., 2011, Trazzi et al., 2013). The outcome of this over-inhibition was impairment of neurogenesis and neurite development. Treatments that restored Shh signaling reverted both these defects. In agreement with a key role played by AICD in neurogenesis alterations in the Ts65Dn model, it has been shown that AICD transgenic mice exhibit impaired neurogenesis, similarly to trisomic mice (Ghosal et al., 2010).

The evidence reported above suggests that the impairment of the Shh pathway that is due to APP-AICD-dependent Ptch1 over-expression may be a key mechanism that underlies reduced proliferation and impaired maturation of neuronal precursors in the trisomic brain. Since Ptch1 over-expression keeps the pathway under repression, therapies have been attempted with SAG, a drug that activates the Shh pathway by acting downstream of Ptch1 (Das et al., 2013, Roper et al., 2009). Though this strategy is effective, the use of activators of the Shh pathway may pose some caveats because the Shh pathway is implicated in the development of cancers (Katoh and Katoh, 2009). Since Ptch1 over-expression in the DS brain is due to excessive AICD levels, an ideal approach to restore Ptch1 levels and, hence, Shh signaling, would be to reduce AICD formation through inhibitors of γ-secretase. During the last few years, various selective APP γ-secretase inhibitors have been developed (Basi et al., 2010, Fleisher et al., 2008, Probst et al., 2013) as strategic tools to reduce Aβ levels in Alzheimer's disease. So far, no study has explored the possibility to exploit γ-secretase inhibitors as a pharmacological tool to reduce the excessive levels of AICD that characterize the DS brain. Reduction of AICD levels is expected to normalize Ptch1 levels, Shh signaling and, ultimately, neurogenesis. Based on this rationale, the goal of the current study was to establish whether treatment with a selective γ-secretase inhibitor positively impacts neurogenesis in the trisomic brain. To clarify this issue we have used the Ts65Dn mouse, a widely-used model of DS that exhibits several features of the human condition. Here we provide evidence that a selective γ-secretase inhibitor restores Shh signaling in trisomic neural precursor cells and that this effect leads to restoration of neurogenesis and hippocampal development.

## 2 Methods

### 2.1 Colony

Ts65Dn mice were generated by mating B6EiC3Sn a/ATs(17 < 16 >)65Dn females with C57BL/6JEiJ x C3H/HeSnJ (B6EiC3Sn) F1 hybrid males. This parental generation was provided by Jackson Laboratories (Bar Harbour, ME, USA). To maintain the original genetic background, the used mice were the first generation of this breeding. Animals were karyotyped as previously described (Reinholdt et al., 2011). The day of birth was designated postnatal day zero (P0). A total of 49 mice were used. The animals' health and comfort were controlled by the veterinary service. The animals had access to water and food ad libitum and lived in a room with a 12:12 h dark/light cycle. Experiments were performed in accordance with the Italian and European Community law for the use of experimental animals and were approved by Bologna University Bioethical Committee (Prot. N.28-IX/9). In this study all efforts were made to minimize animal suffering and to keep the number of animals used to a minimum.

### 2.2 In vitro experiments

#### 2.2.1 Cell cultures

Cells were isolated from the SVZ of newborn (P2) euploid (n = 5) and Ts65Dn (n = 6) mice and neurosphere cultures were obtained as previously reported (Trazzi et al., 2011, Trazzi et al., 2013). Cells were cultured in suspension in Dulbecco's modified Eagle's medium (DMEM)/F12 (1:1) containing B27 supplements (2%), basic fibroblast growth factor (FGF-2, 20 ng/ml), epidermal growth factor (EGF, 20 ng/ml), heparin (5 μg/ml) and antibiotics (penicillin: 100 units/ml; streptomycin: 100 mg/ml). Primary neurospheres were dissociated at days 7–8 using Accutase (PAA, Pasching, Austria) to derive secondary neurospheres. The sub-culturing protocol consisted of neurosphere passaging every 7 days with whole culture media change (with freshly added FGF-2 and EGF). All experiments were done using neurospheres obtained after one to three passages from the initially prepared cultures. Cell cultures were kept in a 5% CO_2_ humidified atmosphere at 37 °C.

#### 2.2.2 BrdU immunocytochemistry in neurospheres

For proliferation analysis, neurospheres were dissociated in a single cell suspension and plated onto poly-l-ornithine-coated 24-well chamber slides at a density of 3 × 10^4^ cells per well in a DMEM/F-12 medium containing EGF (20 ng/ml), FGF (20 ng/ml) and 2% FBS. Cells were treated with ELN 1 nM and, in some experiments, with 10 μg/ml cyclopamine hydrate (Sigma) for 24 h. Cells were then treated with BrdU for additional 6 h, paraformaldehyde fixed and stained with a mouse anti-5-bromo-2-deoxyuridine (BrdU) monoclonal antibody (1:100; Roche Applied Science) and a Cy3-conjugated anti-mouse secondary antibody (1:200; Sigma). Samples were counterstained with Hoechst-33258. Digital images were captured using an Eclipse TE 2000-S microscope and the NIS-Elements AR software (Nikon).

#### 2.2.3 In vitro analysis of differentiation and neurite length of NPCs

Primary neurospheres were dissociated and plated on cover slips coated with 15 mg/ml poly-l-ornithyine (Sigma). Cells were grown for 2 days in a DMEM/F-12 medium containing EGF (20 ng/ml), FGF (20 ng/ml), and 2% FBS and then transferred to a differentiating medium (EGF and FGF free plus 1% fetal bovine serum) for 5 days. Every 2 days, half of the medium was replenished with fresh differentiating medium. ELN 1 nM was administered on alternate days throughout the differentiation period (6 days) starting from the first day. Differentiated cells were fixed (see above) and incubated with anti-glial fibrillary acidic protein (1:400; GFAP mouse monoclonal, Sigma) and anti-β-III tubulin (1:100; rabbit polyclonal, Sigma), as primary antibodies, and with anti-mouse FITC-conjugated (1:200; Sigma) and anti-rabbit Cy3-conjugated (1:200; Jackson Laboratories), as secondary antibodies. Samples were counterstained with Hoechst-33258. Cells were counted in five different fields of each cover slip. Number of positive cells for each antibody was referred to the total number of Hoechst-stained nuclei. Evaluation of total neurite length of cells differentiated into neurons was performed by using the image analysis system Image-Pro Plus (Media Cybernetics, Rockville, MD, USA).

#### 2.2.4 Western blotting

Total proteins were obtained from neurospheres as previously described (Trazzi et al., 2011). Protein concentration was estimated by the Lowry method. Proteins (50 μg) were subjected to electrophoresis on a 4–12% NuPAGE Bis-Tris Precast Gel (Novex, Life Technologies, Ltd, Paisley, UK) and transferred to a Hybond ECL nitrocellulose membrane (Amersham Life Science). The following primary antibodies were used: anti-Amyloid Precursor Protein, C-Terminal (anti-APP; 1:5000; Sigma); anti-Ptch1 rat monoclonal (1:1000; Abcam), anti-GSK3β mouse mAb (anti-GSK3β; 1:1000; Cell Signaling Technology); anti-Phospho-GSK3β (Ser9) XP® Rabbit mAb (anti-pGSK3β; 1:1000; Cell Signaling Technology); and anti-GAPDH produced in rabbit (anti-GAPDH; 1:5000; Sigma). For AICD detection, the membrane was processed for antigen-retrieval as previously described (Trazzi et al., 2011). Densitometric analysis of digitized images was performed with the software Image-Pro Plus and intensity for each band was normalized to the intensity of the corresponding GAPDH band.

### 2.3 In vivo experiments

#### 2.3.1 Experimental protocol

During the last few years, various selective APP γ-secretase inhibitors have been developed by ELAN Inc. (Basi et al., 2010), as strategic tools to reduce Aβ levels in Alzheimer's disease. Among these, ELND006 is a γ-secretase inhibitor which retains selectivity and incorporates improved drug-like properties (Basi et al., 2010, Probst et al., 2013). Euploid (n = 11) and Ts65Dn (n = 11) mice received a daily subcutaneous injection of ELND006 (ELN; gift by ELAN Inc, USA) dissolved in 25% PEG300, 25% ethylene glycol, 25% cremophor, 15% ethanol, 10% propanol from postnatal day 3 (P3) to postnatal day 15 (P15). Based on previous evidence, we used a daily dose of 30 mg/kg (Basi et al., 2010, Brigham et al., 2010, Probst et al., 2013). Age-matched euploid (n = 8) and Ts65Dn (n = 8) mice were injected with the vehicle. Each treatment group had approximately the same composition of males and females. Animals (4–7 animals for each condition) received a subcutaneous injection (150 μg/g body weight) of BrdU (5-bromo-2-deoxyuridine; Sigma), a marker of proliferating cells (Nowakowski et al., 1989) in Tris HCl 50 mM (at 11–12 am) 2 h before being euthanized. The brain of the other animals (4 animals for each condition) was quickly removed, the hippocampal formation was dissected, kept at − 80 °C and used for western blotting.

#### 2.3.2 Histological procedures

Mice that had received BrdU were deeply anesthetized, the brain was removed cut along the midline. The left hemisphere was fixed by immersion in Glyo-Fixx as previously described (Bianchi et al., 2010) and the right hemisphere was fixed in 4% paraformaldehyde and frozen. The left hemisphere was embedded in paraffin and cut in series of 8-μm-thick coronal sections that were attached to poly-lysine coated slides and used for BrdU, Ki-67, and cleaved caspase-3 immunohistochemistry and for hematoxylin staining. The right hemisphere was cut with a freezing microtome in 30-μm-thick coronal sections that were serially collected in PBS and used for synaptophysin (SYN) and postsynaptic density protein-95 (PSD-95) immunohistochemistry.

##### 2.3.2.1 BrdU immunohistochemistry

One out of 20 sections was taken from the beginning of the lateral ventricle to the end of the hippocampal formation (n = 14–18 sections). Sections were processed as previously described (Bianchi et al., 2010) and incubated overnight at 4 °C with a primary antibody anti-BrdU (mouse monoclonal 1:100, Roche Applied Science, Mannheim, Germany). Detection was performed with an HRP-conjugated anti-mouse secondary antibody (dilution 1:200; Jackson Immunoresearch, West Grove, PE, USA) and DAB kit (Vector Laboratories, Burlingame, CA, USA).

##### 2.3.2.2 Ki-67 immunohistochemistry

One out of 20 sections was taken starting from the beginning of the lateral ventricle up to the end of the hippocampal formation (n = 16–20 sections). Sections were incubated overnight at 4 °C with rabbit monoclonal anti-Ki67 antibody (1:100; Thermo Scientific). Detection was performed with a HRP-conjugated anti-rabbit secondary antibody (1:200; Jackson Immunoresearch) and DAB kit (Vector Laboratories).

##### 2.3.2.3 Cleaved caspase–3 immunohistochemistry

One out of 20 sections from the beginning of the lateral ventricle to the end of the hippocampal formation (n = 16–20 sections) was processed for cleaved caspase-3 immunohistochemistry, as previously described (Bianchi et al., 2010).

##### 2.3.2.4 SYN and PSD-95 immunohistochemistry

Free-floating sections (n = 4–6 per animal) taken at the level of the hippocampal formation were submitted to fluorescence immunohistochemistry for SYN and PSD-95. Sections were counterstained with Hoechst dye in order to label cell nuclei. Sections were incubated for 48 h at 4 °C with mouse monoclonal anti-SYN (SY38) antibody (Millipore-Biomanufacturing and Life Science Research, Billerica, MA, USA) or rabbit polyclonal anti-PSD-95 antibody (Abcam) both diluted 1:1000. Sections were then incubated overnight at 4 °C with a FITC-conjugated goat anti-mouse antibody or with a CY3-conjugated anti-rabbit (Jackson Laboratory) antibody both diluted 1: 200.

##### 2.3.2.5 Hematoxylin-staining

One out of 20 sections, taken from the beginning to the end of the hippocampal formation (n = 9–12) was stained with hematoxylin.

#### 2.3.3 Measurements

##### 2.3.3.1 Number of BrdU, Ki-67, and cleaved caspase-3 positive cells

Cells were sampled from the dentate gyrus (DG) and the rostral subventricular zone (SVZ). The total number of positive cells in the DG and SVZ was estimated by multiplying the total number counted in the series of sampled sections by the inverse of the section sampling fraction (ssf = 1/20).

##### 2.3.3.2 Synaptic terminals

The intensity of SYN and PSD-95 immunoreactivity in the molecular layer of the DG and in the stratum lucidum of field CA3 was determined by the optical densitometry of immunohistochemically-stained sections. Fluorescence images were captured using a Nikon Eclipse E600 microscope equipped with a Nikon Digital Camera DXM1200 (ATI system). Densitometric analysis of SYN or PSD-95 was carried out using the software Image Pro Plus as previously described (Guidi et al., 2013). Images immunoprocessed for SYN or PSD-95 were acquired with a confocal microscope (Nikon Ti-E fluorescence microscope coupled with an A1R confocal system, Nikon). In each section three images from the regions of interest indicated above were captured and the density of individual puncta exhibiting SYN or PSD-95 immunoreactivity was evaluated as previously described (Guidi et al., 2013, Stagni et al., 2013).

##### 2.3.3.3 Stereology of the DG

The volume of the granule cell layer was evaluated in hematoxylin stained sections. The volume of the granule cell layer (V_ref_) was estimated (West and Gundersen, 1990) by multiplying the sum of the cross-sectional areas by the spacing T between sampled sections (T = 160 μm). In the series of sections from the DG counterstained with the Hoechst dye we evaluated granule cell numerical density using the optical fractionator. Counting frames with a side length of 30 μm and a height of 8 μm spaced in a 150 μm square grid (fractionator) were used. Granule cell nuclei intersecting the uppermost focal plane and intersecting the exclusion lines of the count frame were not counted. The neuron density (Nv) is given by

$$N_{V}=\left(\sum Q/\sum \mathrm{dis}\right)/\mathrm{Vdis}$$

where Q is the number of particles counted in the disectors, dis is the number of disectors and Vdis is the volume of the disector. The total number (N) of granule cells was estimated as the product of V_ref_ and the numerical density (Nv)

$$N=N_{V}\times V_{ref}\text{.}$$

#### 2.3.4 Western blotting

The same western blot analyses carried out in vitro were carried out in hippocampal homogenates from treated and untreated mice.

### 2.4 Statistical analysis

Results are presented as the mean ± SE of the mean. Statistical testing was performed with ANOVA followed by post hoc comparisons with Duncan's test. A probability level of p < 0.05 was considered to be statistically significant.

## 3 Results

### 3.1 Effect of treatment with ELN on proliferation, phenotype acquisition, and maturation of trisomic NPCs

**Fig. 1:**
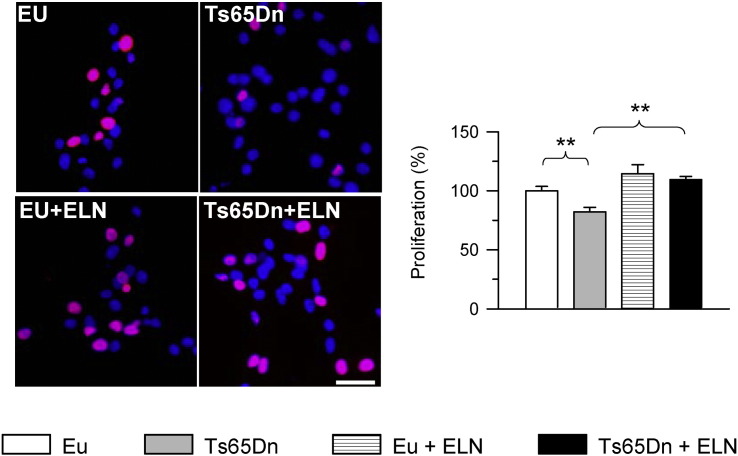
Effect of APP γ-secretase inhibition on proliferation of trisomic neural precursor cells. Neural precursor cells (NPCs) from the SVZ of euploid and Ts65Dn mice were treated with an inhibitor of APP γ-secretase (ELND006) 1 nM for 24 h. Cells were exposed to BrdU for 6 h in order to label proliferating cells. Images show examples of BrdU-labeled cells (red) in untreated and treated cultures. Nuclei were counterstained with Hoechst staining (blue). Scale bar = 20 μm. The histograms show the number (mean ± SE) of proliferating cells expressed as percentage of untreated euploid NPCs. ** p < 0.01 (Duncan's test after ANOVA). Abbreviations: ELN, ELND006; Eu, euploid.

**Fig. 2:**
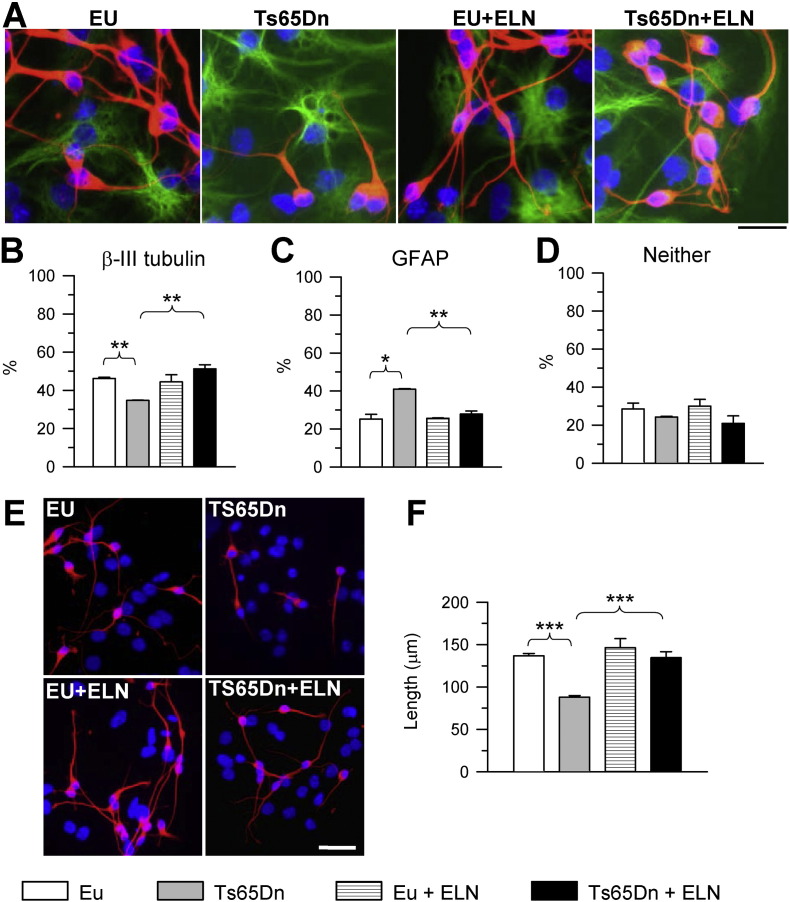
Effect of APP γ-secretase inhibition on differentiation of trisomic neural precursor cells. NPCs from the SVZ of euploid and Ts65Dn mice were treated with an inhibitor of APP γ-secretase (ELND006) 1 nM during differentiation for seven days in culture. A: Examples of differentiated cells expressing the early neuronal marker β-III tubulin (red) and the astrocytic marker GFAP (green) in untreated and treated cultures. Nuclei were counterstained with Hoechst staining (blue). Scale bar = 20 μm. B–D: Percentage of β-III tubulin-positive cells (B), GFAP-positive cells (C), and cells with an undetermined phenotype (D; neither) in untreated and treated cultures. E: Examples of cells expressing the early neuronal marker β-III tubulin in untreated and treated cultures. Note that euploid cells exhibit longer neuritic processes in comparison with trisomic cells. Nuclei were counterstained with Hoechst staining (blue). Scale bar = 50 μm. F: Mean length of neurite processes per cell. Histograms in B–D and F are mean ± SE. * p < 0.05; ** p < 0.01; p < 0.001 (Duncan's test after ANOVA). Abbreviations: ELN, ELND006; Eu, euploid.

Neuronal precursor cells (NPCs) from the subventricular zone (SVZ) of neonate Ts65Dn mice exhibit defects in proliferation rate, neuronal phenotype acquisition, and maturation (Trazzi et al., 2011, Trazzi et al., 2013). In order to establish the effect of γ-secretase inhibition on these defects, NPCs from the SVZ of Ts65Dn and euploid neonate (P2) mice were grown as neurospheres and treated with ELN. ELND006 inhibits γ-secretase mediated cleavage of APP in an in vitro enzyme assay with an IC50 of 0.3 nM and inhibits Aβ production in cells with an IC50 of approximately 1 nM. In pilot experiments we tested the effects of different doses of ELN (0.1, 1.0 and 10.0 nM) on neurosphere proliferation. The lowest dose had no effect on proliferation, the two higher doses increased cell proliferation in a similar manner in trisomic neurospheres, though in some samples the highest dose induced a smaller increase in proliferation in comparison with the intermediate dose. Therefore, we treated cultures with the dose that induced the higher increase in proliferation rate in trisomic neurospheres (1.0 nM). We found that while untreated trisomic NPCs exhibited a notably reduced number of proliferating cells, assessed with BrdU immunohistochemistry, treated cultures exhibited a number of proliferating cells similar to that of untreated euploid cultures (Fig. 1), indicating that treatment had restored the proliferation defect that characterizes trisomic NPCs. Treatment with ELN had no effect on proliferation rate in euploid NPCs (Fig. 1). We next examined the phenotype acquired by untreated and treated differentiating euploid and trisomic NPCs. Consistently with previous evidence (Trazzi et al., 2013), untreated trisomic NPCs exhibited a reduction in the acquisition of a neuronal phenotype, as shown by β-III tubulin immunoreactivity, and an increase in the acquisition of an astrocytic phenotype, as shown by GFAP immunoreactivity (Fig. 2A–D). After treatment with ELN this defect was fully rescued and in treated trisomic NPCs the percentage of new neurons and new astrocytes became similar to that of untreated euploid NPCs (Fig. 2A–D). Trisomic NPCs, in addition to proliferation impairment, exhibit defective development of neuritic processes (Trazzi et al., 2013). In order to establish whether treatment positively impacts neurite development, we evaluated the neuritic length of NPCs treated with ELN during differentiation. Treatment with ELN rescued defective neurite development of trisomic NPCs and total neurite length became similar to that of untreated euploid NPCs (Fig. 2E, F). Treatment with ELN had no effect on neurite development in euploid NPCs (Fig. 2E, F).

### 3.2 Effect of treatment with ELN on APP, AICD, Ptch1 and GSK3β protein expression in trisomic NPCs

**Fig. 3:**
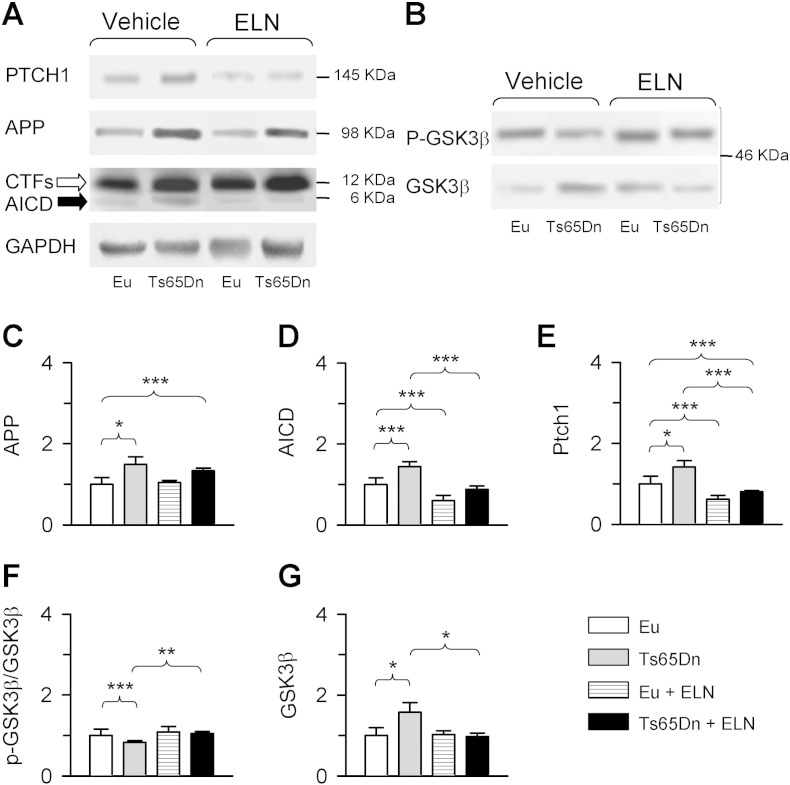
Effect of APP γ-secretase inhibition on APP, AICD, Ptch1 and GSK3β protein expression in trisomic NPCs. Neural precursor cells (NPCs) from the SVZ of euploid and Ts65Dn mice, grown as neurospheres, were treated with an inhibitor of APP γ-secretase (ELND006) 1 nM for 24 h. A, B: Western blot analysis of Ptch1, APP, CTFs, AICD and GAPDH (A) and p-GSK3β and GSK3β (B) in neurospheres from untreated and treated euploid and Ts65Dn mice. The empty and filled arrows in (A) indicate the CTFs and AICD band, respectively. Note the low levels of AICD in comparison with the high levels of the CTFs. Western blots in (B) are from the same brain samples as in (A). C–G: The histograms (mean ± SE) show APP (C), AICD (D), Ptch1 (E), pGSK3β (F) and GSK3β (G) levels expressed as fold difference in comparison with untreated euploid NPCs. Data in (C–E, G) were normalized to GAPDH and data in (F) were normalized to total GSK3β. Western blot images are explicative reconstructions. * p < 0.05; ** p < 0.01; *** p < 0.001 (Duncan's test after ANOVA). Abbreviations: ELN, ELND006; Eu, euploid.

Evaluation of APP and AICD levels showed that untreated trisomic NPCs had higher levels of APP (Fig. 3A, C) and AICD (Fig. 3A, D) in comparison with untreated euploid NPCs. In agreement with the inhibitory action exerted by ELN on γ-secretase activity, in trisomic NPCs treated with ELN there was a reduction in AICD levels that became similar to those of euploid NPCs (Fig. 3A, D). The counterpart of this effect was a parallel increase in the levels of APP CTFs (Fig. 3A). Since in trisomic NPCs high AICD levels cause an increase in the transcription of Ptch1 (Trazzi et al., 2011), normalization of AICD levels should lead to a reduction in Ptch1 expression. We found that the abnormally high levels of Ptch1 in trisomic NPCs were reduced by treatment with ELN and became lower in comparison with untreated euploid NPCs (Fig. 3A, E). A reduction in AICD and Ptch1 levels also took place in treated euploid NPCs (Fig. 3A, D, E). These findings indicate that inhibition of APP γ-secretase reinstates the abnormally high AICD and Ptch1 levels that characterize trisomic NPCs.

GSK3β, a kinase involved in neurogenesis and neuron maturation (Kim and Snider, 2011), has been shown to have enhanced activity in the trisomic brain (Trazzi et al., 2014). This kinase becomes active when dephosphorylated, thereby negatively influencing proliferation and differentiation of neural precursor cells. AICD has been shown to act in the cytoplasmic domain and to reduce the phosphorylation of GSK3β at Ser9 (Zhou et al., 2012), thereby increasing its activity. Evaluation of the levels of the phosphorylated form of GSK3β in comparison to total GSK3β showed that in untreated trisomic NPCs the phosphorylated form was reduced in comparison with the euploid counterparts (Fig. 3B, F). In treated trisomic NPCs there was an increase in the levels of the phosphorylated form that became similar to those of untreated euploid NPCs (Fig. 3B, F). AICD, in addition to modulate phosphorylation of GSK3β, transcriptionally regulates its expression (Cao and Sudhof, 2001, Nalivaeva and Turner, 2013, von Rotz et al., 2004). We found that untreated trisomic NPCs had higher levels of GSK3β in comparison with the euploid counterparts (Fig. 3B, G). In treated trisomic NPCs there was a large reduction in GSK3β levels that became similar to those of untreated euploid NPCs (Fig. 3B, G). Unlike in trisomic neurospheres, in euploid neurospheres treatment did not change levels of phosphorylated GSK3β and total GSK3β levels (Fig. 3F, G). However a 13-day-treatment affected levels of GSK3β and of phosphorylated GSK3β in euploid mice (see Fig. 8D, E), suggesting that a prolonged treatment is necessary in the euploid condition.

### 3.3 Inhibition of Shh signaling prevents the pro-neurogenic effect of ELN in trisomic NPCs

Binding of the Shh peptide to Ptch1 alleviates its inhibition of Smoothened (Smo), leading to Smo phosphorylation and activation of the pathway (Zhang et al., 2004). The data reported above suggested that the positive effect of treatment with ELN on neurogenesis was due to normalization of Shh signaling due to restoration of Ptch1 levels and, hence, disinhibition of Smo. In order to verify this hypothesis, we treated trisomic and euploid NPCs with ELN and/or cyclopamine, an inhibitor of Smo. If the effect of ELN on neurogenesis were mediated by the Shh pathway, inhibition of Smo with cyclopamine should prevent the effects of ELN on proliferation.

**Fig. 4:**
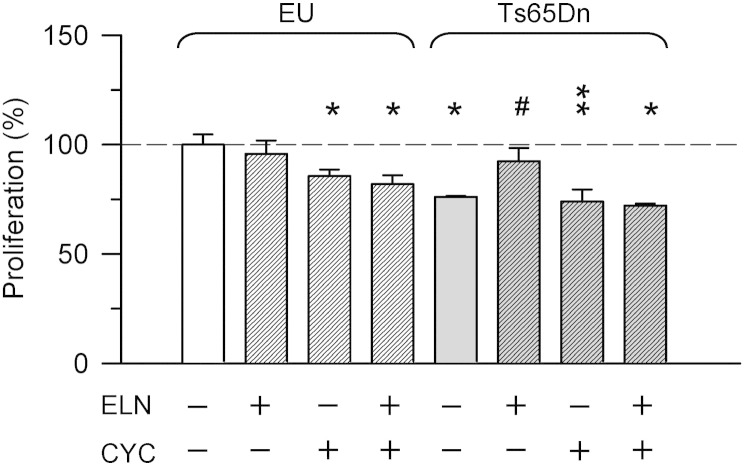
Inhibition of the Shh pathway prevents the effects of ELN on proliferation. Neural precursor cells (NPCs) from the SVZ of euploid (EU) and Ts65Dn mice, grown as neurospheres, were treated with an inhibitor of APP γ-secretase (ELND006; ELN) 1 nM, cyclopamine (CYC) 10 μg/ml, and ELN plus cyclopamine for 24 h. Cells were exposed to BrdU for 6 h in order to label proliferating cells. The histogram shows the number (mean ± SE) of proliferating cells expressed as percentage of untreated euploid NPCs. The asterisks indicate a difference in comparison with untreated euploid neurospheres. The symbol # indicates a difference in comparison with untreated trisomic neurospheres. * p < 0.05; ** p < 0.01; # p < 0.05 (Duncan's test after ANOVA).

We found that treatment with cyclopamine alone reduced the proliferation rate of euploid NPCs (Fig. 4A). This confirms basal activation of the Shh pathway in cultures of NPCs due to endogenous production of Shh (Osterberg and Roussa, 2009, Trazzi et al., 2011). Unlike in euploid NPCs, in trisomic NPCs cyclopamine had no effect on proliferation rate (Fig. 4A). While in trisomic NPCs treated with ELN there was an increase in proliferation rate, in trisomic NPCs treated with ELN plus cyclopamine this increase did not take place and the proliferation rate remained at the typically low levels of untreated trisomic NPCs (Fig. 4A). The finding that in trisomic NPCs treatment normalizes Ptch1 levels (Fig. 3A,E) and that inhibition of the Shh pathway through cyclopamine prevents restoration of proliferation (Fig. 4A) strongly suggests that the increase in proliferation rate induced by treatment with ELN is mediated through the Shh pathway.

### 3.4 Effect of neonatal treatment with ELN on neurogenesis in the SVZ and SGZ of Ts65Dn mice

**Fig. 5:**
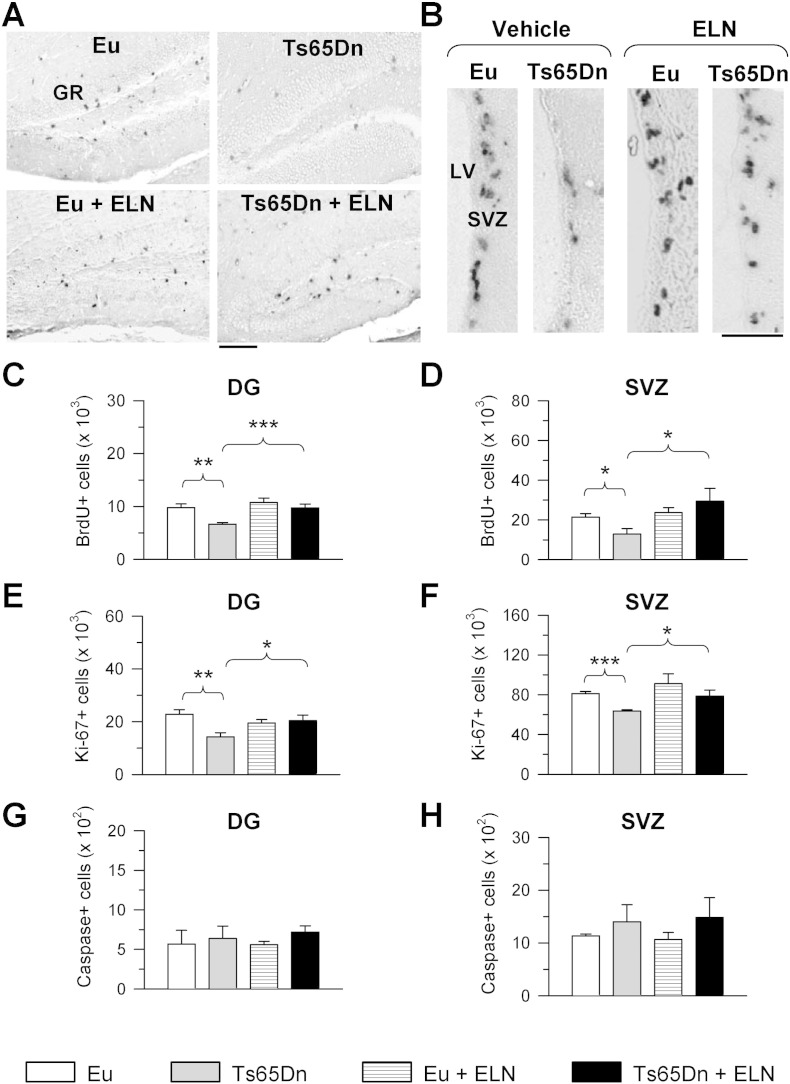
Effect of APP γ-secretase inhibition on proliferation potency in the dentate gyrus and subventricular zone of Ts65Dn and euploid mice. Animals received a daily injection of ELND006 or vehicle in the period P3–P15. On P15 they received one injection of BrdU and were sacrificed after 2 h. A–B: Sections immunostained for BrdU from the DG (A) and SVZ (B) of untreated and treated euploid and Ts65Dn mice. Scale bar: 100 μm (A); 50 μm (B). C, D: Total number of BrdU-positive cells in the DG (C) and SVZ (D) of untreated euploid (n = 5) and Ts65Dn (n = 4) mice and euploid (n = 4) and Ts65Dn (n = 5) mice treated with ELN. E, F: Number of Ki-67 positive cells in the dentate gyrus (E) and SVZ (F) of untreated euploid (n = 5) and Ts65Dn (n = 4) mice and euploid (n = 4) and Ts65Dn (n = 5) mice treated with ELN. Number of cleaved caspase-3 positive cells in the DG (E) and SVZ (F) of untreated euploid (n = 5) and Ts65Dn (n = 4) mice and euploid (n = 4) and Ts65Dn (n = 5) mice treated with ELN. Values (mean ± SE) in (C–H) represent totals for one hemisphere. * p < 0.05; ** p < 0.01; *** p < 0.001 (Duncan's test after ANOVA). Abbreviations: DG, dentate gyrus; ELN, ELND006; Eu, euploid; LV, lateral ventricle; SVZ, subventricular zone.

Results obtained in vitro suggested that treatment with ELN might be used for correcting the neurogenesis defects that characterize DS. Therefore, we treated Ts65Dn (and euploid) mice with ELN from postnatal day 3 to postnatal day 15, in order to establish whether treatment was able to restore neurogenesis in vivo. At postnatal day 15 mice were injected with BrdU, a marker of the S-phase of the cell cycle, and were killed after 2 h. Evaluation of the number of proliferating cells in SGZ of the dentate gyrus (DG) and in the SVZ of the lateral ventricle showed that, consistently with previous evidence (Bianchi et al., 2010), Ts65Dn mice had fewer BrdU + cells in comparison with euploid mice (Fig. 5A–D). Treatment with ELN increased the number of BrdU + cells in Ts65Dn mice, so that treated mice had a similar number of BrdU + cells as untreated euploid mice both in the DG (Fig. 5C) and SVZ (Fig. 5D). A comparison between treated and untreated euploid mice showed no effect of treatment (Fig. 5C,D). To establish whether treatment with ELN restores the overall size of the population of neural precursors in the DG and SVZ of Ts65Dn mice, we evaluated the number of cells immunopositive for Ki-67, an endogenous marker expressed during phases G_1_, S, G_2_ and M of the cell cycle (Scholzen and Gerdes, 2000). We found that in the DG of untreated Ts65Dn mice there were fewer Ki-67 + cells in comparison with euploid mice (Fig. 5E). In treated Ts65Dn mice the number of cycling cells underwent an increase and became similar to that of euploid mice (Fig. 5E). Similar findings were obtained in the SVZ (Fig. 5F). The protein cleaved caspase-3 is one of the hallmarks of apoptotic death (Blomgren et al., 2007). Evaluation of the number of cells expressing cleaved caspase-3 showed that untreated Ts65Dn mice had a similar number of apoptotic cells as untreated euploid mice both in the DG and SVZ and that the number of apoptotic cells was not affected by treatment both in euploid and Ts65Dn mice (Fig. 5G,H). Taken together, these results indicate that treatment with ELN during the first two postnatal weeks restores the number of neural precursor cells in the DG and SVZ of Ts65Dn mice and suggest that this effect is due to an increase in proliferation potency and not to a decrease in cell death.

### 3.5 Effect of neonatal treatment with ELN on granule cell number in the dentate gyrus of Ts65Dn mice

**Fig. 6:**
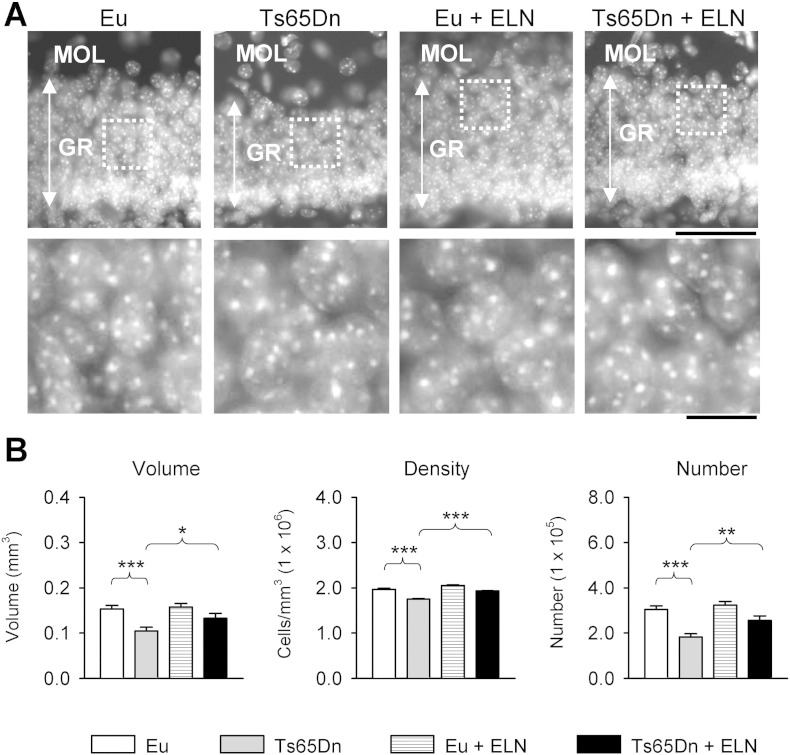
Effect of APP γ-secretase inhibition on total granule cell number in the dentate gyrus. A: Hoechst-stained coronal section across the dentate gyrus of an animal of each experimental group. The higher magnification images in the lower row correspond to the region enclosed in the dotted box in the upper row. Scale bar = 50 μm (upper row); 10 μm (lower row). B: Volume of the granule cell layer, density of granule cells (number per mm^3^) and total number of granule cells of the DG. Values are mean ± SE. Volume and granule cell number refer to one hemisphere. * p < 0.05; ** p < 0.01; *** p < 0.001 (Duncan's test after ANOVA). Abbreviations: ELN, ELND006; Eu, euploid; GR, granule cell layer; MOL, molecular layer.

In mice, the SGZ produces most of the granule cells that populate the granule cell layer of the DG in the first two postnatal weeks (Altman and Bayer, 1975). Hypocellularity in the dentate gyrus of Ts65Dn mice due to proliferation impairment has been documented starting from neonatal life stages (Guidi et al., 2014, Lorenzi and Reeves, 2006). In order to establish whether the increase in the number of neural cell precursors in the DG of treated Ts65Dn mice (Fig. 5) translated into restoration of total granule cell number, we evaluated the number of granule cells at the end of treatment. In euploid mice, treatment did not change the volume of the granule cell layer, granule cell density and total granule cell number (Fig. 6). In contrast, in Ts65Dn mice, treatment significantly increased the volume of the granule cell layer, granule cell density and total granule cell number (Fig. 6). A comparison between treated Ts65Dn and untreated euploid mice showed no differences (Fig. 6), indicating a treatment-induced rescue in the development of the granule cell layer.

### 3.6 Effect of neonatal treatment with ELN on hippocampal synapse development

In view of restoration of total granule cell number in treated Ts65Dn mice we wondered whether this effect was accompanied by restoration of synapse development, a process that is impaired in the trisomic brain (Belichenko et al., 2007, Chakrabarti et al., 2007, Guidi et al., 2013, Kurt et al., 2004). To this purpose, in hippocampal sections from treated and untreated mice we evaluated the immunoreactivity for synaptophysin (SYN), a marker of presynaptic terminals, and the postsynaptic density protein-95 (PSD-95), a marker of postsynaptic sites. We focused on the molecular layer of the DG because this layer receives the major input to the hippocampus, the perforant pathway, and the stratum lucidum of field CA3, the site of termination of the axons of the granule cells (Amaral and Witter, 1995).

**Fig. 7:**
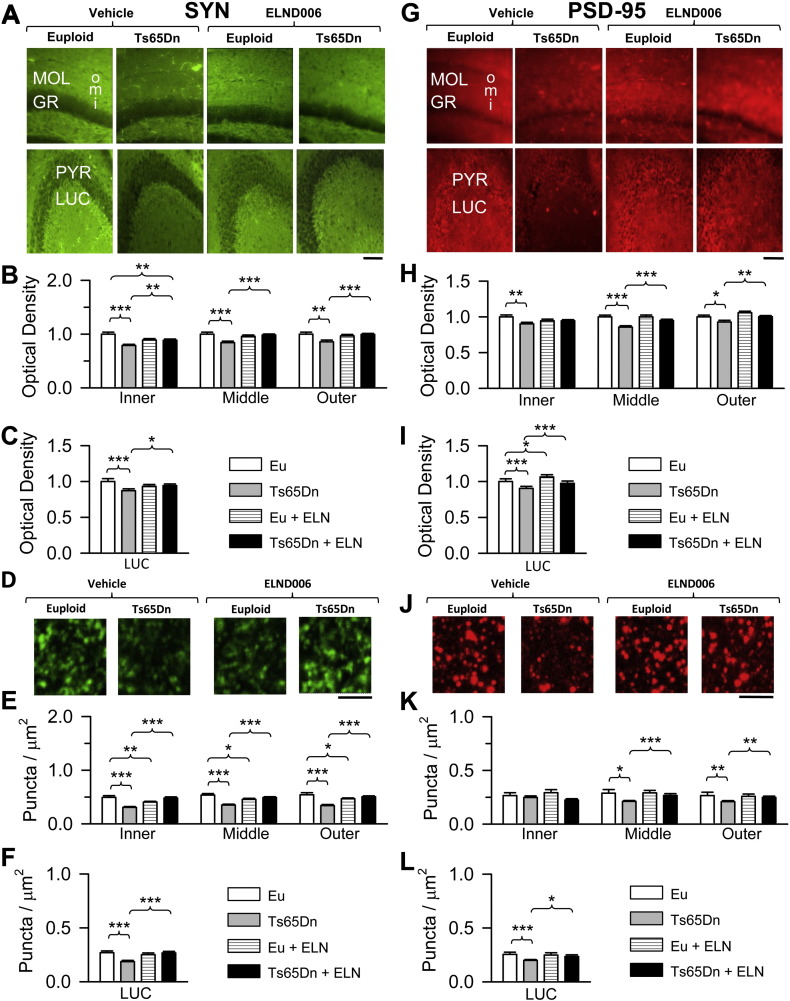
Effect of APP γ-secretase inhibition on synapse development in the hippocampal formation. A, G: Sections processed for SYN (A) and PSD-95 (G) immunofluorescence from the DG (upper row) and CA3 (lower row) of an animal from each experimental group. Scale bar = 100 μm. B, C, H, I: Optical density of SYN (B, C) and PSD-95 (H, I) immunoreactivity in the inner, middle and outer third of the molecular layer of the DG (B, H) and the stratum lucidum of CA3 (C, I) of untreated euploid (n = 6) and Ts65Dn (n = 5) mice and euploid (n = 6) and Ts65Dn (n = 5) mice treated with ELND006. For each region, data of SYN and PSD-95 immunoreactivity are given as fold difference vs. untreated euploid mice. D, J: Images, taken with the confocal microscope, of sections processed for SYN (D) and PSD-95 (J) immunofluorescence from the DG of an animal of each experimental group. Scale bar = 3 μm. E, F, K, L: Number of puncta per μm^2^ exhibiting SYN (E, F) and PSD-95 (K, L) immunoreactivity in the inner, middle and outer third of the molecular layer of the DG (E, K) and the stratum lucidum of CA3 (F, L) of untreated euploid (n = 6), untreated Ts65Dn (n = 5), and euploid (n = 6) and Ts65Dn (n = 5) mice treated with ELND006. Values in B, C, E, F, F, H, I, K and L represent mean ± SE. * p < 0.05; ** p < 0.01; *** p < 0.001 (Duncan's test after ANOVA). Abbreviations: ELN, ELND006; Eu, euploid; GR, granule cell layer; i, inner; LUC, stratum lucidum; m, middle; MOL, molecular layer; o, outer.

Results showed that in untreated Ts65Dn mice the immunoreactivity for SYN was significantly lower than in untreated euploid mice in the inner (− 21%), middle (− 16%) and outer (− 15%) molecular layer of the DG (Fig. 7A, B) and in the stratum lucidum (− 13%) of CA3 (Fig. 7A, C). In parallel with the reduction of SYN immunoreactivity, in Ts65Dn mice there was a reduction in the immunoreactivity for PSD-95 in the inner (− 10%), middle (− 15%) and outer (− 8%) molecular layer of the DG (Fig. 7G, H) and in the stratum lucidum (− 10%) of CA3 (Fig. 7G,I). In treated Ts65Dn mice, in all zones of the molecular layer and in the stratum lucidum of CA3 there was an increase in the immunoreactivity for SYN (Fig. 7A, B, C) and PSD-95 (Fig. 7G, H, I) that became similar to that of untreated euploid mice. In euploid mice, treatment did not affect the immunoreactivity for SYN (Fig. 7A, B, C) and PSD-95 (Fig. 7G, H, I) both in the DG and CA3. In order to establish whether the effects of genotype and treatment on protein levels were attributable to different levels of synaptic proteins per synapse or to differences in the number of synapses, we evaluated the density of individual puncta exhibiting either SYN or PSD-95 immunoreactivity. While untreated Ts65Dn mice had fewer puncta exhibiting immunoreactivity for SYN (Fig. 7D, E, F) and PSD-95 (Fig. 7J, K, L) both in the DG and CA3, neonatally-treated Ts65Dn mice had a similar number of SYN (Fig. 7D, E, F) and PSD-95 (Fig. 7J, K, L) puncta as untreated euploid mice in both regions, suggesting that treatment had restored hippocampal synapse development.

### 3.7 Effect of neonatal treatment with ELN on AICD targets in the hippocampus of Ts65Dn mice

**Fig. 8:**
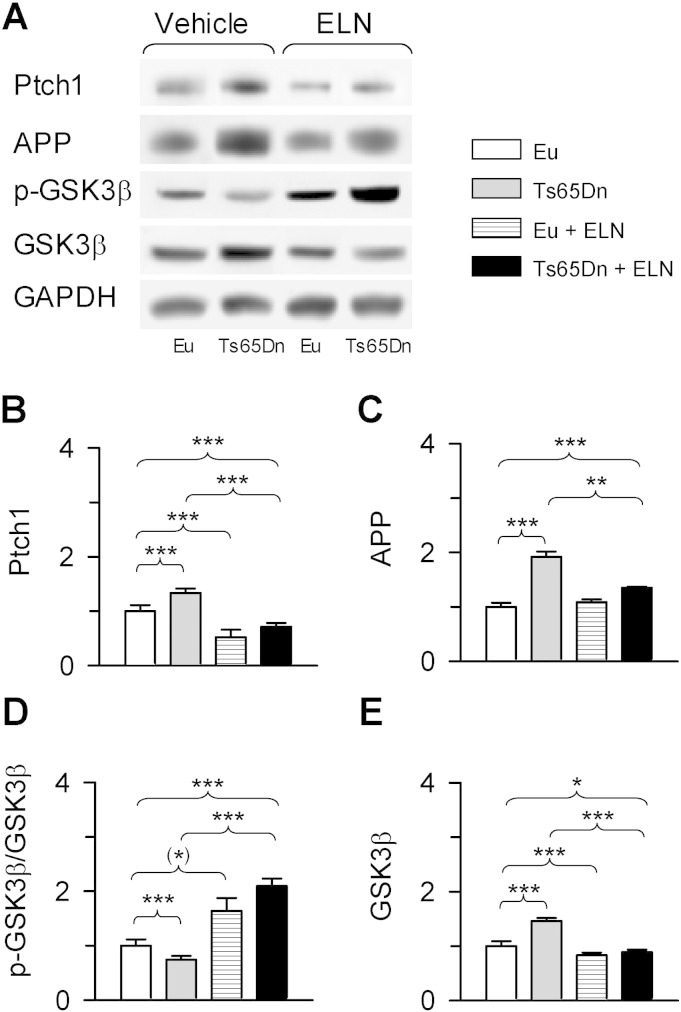
Effect of APP γ-secretase inhibition on AICD targets. A: Western blot analysis of Ptch1, APP, phosphorylated GSK3β and total GSK3β in the hippocampus of Ts65Dn and euploid mice. B-E: Ptch1 (B), APP (C), pGSK3β (D) and total GSK3β (E) levels. Data in B, C and E were normalized to GAPDH and data in (D) were normalized to total GSK3β. Values in (B–E) (mean ± SE) are expressed as fold difference in comparison with untreated euploid mice. All western blot images are explicative reconstructions. (*) p < 0.06; * p < 0.05; ** p < 0.01; *** p < 0.001 (Duncan's test after ANOVA). Abbreviations: ELN, ELND006; Eu, euploid.

Evaluation of Ptch1 expression levels showed that while untreated Ts65Dn mice had higher Ptch1 levels in comparison with euploid mice, after treatment with ELN the levels of Ptch1 underwent a large decrease. A reduction in Ptch1 levels also took place in treated in comparison with untreated euploid mice (Fig. 8A, B). AICD has been shown to transcriptionally up-regulate APP itself (Cao and Sudhof, 2001, Nalivaeva and Turner, 2013, von Rotz et al., 2004). While untreated Ts65Dn mice had notably higher levels of APP in comparison with euploid mice (Fig. 8A, C), in treated Ts65Dn mice APP levels underwent a large reduction in comparison with their untreated counterparts, though they remained larger in comparison with untreated euploid mice (Fig. 8A, C). Treatment had no effect on APP levels in euploid mice (Fig. 8A, C). While in untreated Ts65Dn mice the levels of phosphorylated GSK3β were reduced in comparison with euploid mice (Fig. 8A, D), in treated mice the levels of phosphorylated GSK3β underwent an increase and became even higher than those of untreated euploid mice (Fig. 8A, D). An increase also took place in treated in comparison with untreated euploid mice (Fig. 8A, D). Evaluation of the expression levels of GSK3β showed that while untreated Ts65Dn mice had higher levels of GSK3β in comparison with euploid mice (Fig. 8A, E), in treated Ts65Dn mice GSK3β levels underwent a large reduction (Fig. 8A, E). A reduction in GSK3β levels also took place in treated in comparison with untreated euploid mice (Fig. 8A, E).

## 4 Discussion

### 4.1 Treatment with a γ-secretase inhibitor restores neurogenesis in trisomic NPCs by disinhibiting the Shh pathway

Among the triplicated genes, some located inside and outside the Down syndrome critical region have been reported as likely candidates involved in the neurogenesis impairment that characterizes this genetic condition (Costa and Scott-McKean, 2013, Dierssen, 2012). Previous (Trazzi et al., 2011, Trazzi et al., 2013) and current results highlight the contribution of the triplicated gene APP in neurogenesis impairment in DS. APP triplication causes excessive formation of AICD which, in turn, causes excessive transcription of various genes, including Ptch1, the Shh receptor that inhibits the Shh pathway. This pathway is strongly involved in neural precursor cell proliferation, migration and differentiation (Angot et al., 2008, Machold et al., 2003). We found that treatment with ELN reduced AICD and Ptch1 levels in trisomic NPCs and that this effect was accompanied by neurogenesis restoration. In order to provide a causal link between neurogenesis restoration in trisomic NPCs and the Shh pathway, we used cyclopamine, an inhibitor of Smo. We found that the reduced proliferation rate of trisomic NPCs was not further reduced by cyclopamine alone, suggesting saturation of the inhibition exerted by Ptch1 on Smo. While treatment with ELN restored proliferation of trisomic NPCs, this effect was prevented if the Shh pathway was pharmacologically repressed through cyclopamine. This clearly indicates the involvement of the Shh pathway in the pro-neurogenic effect exerted by treatment. In euploid NPCs cyclopamine alone moderately reduced neurogenesis indicating that the Shh pathway contributes to ongoing neurogenesis under basal conditions. In euploid NPCs ELN did not further increase proliferation, suggesting that reduction of AICD/Ptch1 at levels lower than those normally present in euploid NPCs cannot further enhance the activity of the Shh pathway.

### 4.2 Neonatal treatment with a γ-secretase inhibitor restores neurogenesis in the major postnatal neurogenic niches

Results obtained in vitro were suggestive of a potential impact of treatment with ΕLΝ (or other selective APP γ-secretase inhibitors) as a therapeutic tool for correcting neurogenesis impairment and hypocellularity in the trisomic brain. Since the hippocampal dentate gyrus mainly develops in the early postnatal period in rodents, we decided to treat neonate mice with ELN during the first two postnatal weeks in order to impact hippocampal development. We found that the typically reduced number of neural precursors in the dentate gyrus of Ts65Dn mice was fully normalized at the end of treatment. A similar result was obtained in the SVZ, indicating that treatment positively impacts neurogenic niches located in different brain parts. Treatment did not affect cell death in either neurogenic region, suggesting that there was no adverse effect of treatment on cell survival. Since the bulk of granule neurons is generated during the first 7–10 postnatal days (Altman and Bayer, 1975), we hypothesized that the outcome of neurogenesis restoration in Ts65Dn mice may be an increase in the cellularity of the dentate gyrus. Consistently with this idea, in treated Ts65Dn mice the volume of the granule cell layer and total granule cell number became similar to those of euploid mice, indicating that treatment with ELN leads to normalization of hippocampal cellularity. In euploid mice, neonatal treatment with ELN had no effect on proliferation either in the SVZ or the SGZ, although it reduced Ptch1 levels (see Fig. 8A, B). This suggests that in the normal brain the Shh pathway is optimally active under basal conditions and that a further disinhibition does not increase its efficacy.

A reduced number of pre- and postsynaptic terminals has been documented in various brain regions, including the molecular layer of the DG, of trisomic mice (Belichenko et al., 2007, Chakrabarti et al., 2007, Guidi et al., 2013, Kurt et al., 2004). Therefore, the trisomy-dependent defective functioning of hippocampal circuits appears to be due not only to a reduced number of neurons populating this structure, but also to input–output alterations. The major hippocampal input derives from the entorhinal cortex. Signals then progress to the DG and, consequently, are transferred to field CA3 and then CA1. Signals processed along this circuit are essential for hippocampus-dependent long-term memory functions. We found that treatment restored the number of pre- and postsynaptic terminals in the molecular layer of the DG, suggesting restoration of the major input to the DG. In view of the increase in total granule cell number we sought to establish whether the counterpart of this effect was an increase in the number of pre- and postsynaptic terminals in the stratum lucidum of CA3, the site of termination of the axons of the granule cells. Treated trisomic mice exhibited full restoration of pre- and postsynaptic terminals, suggesting restoration of signal transfer to CA3. Synapse and dendrite development are modulated by various molecular mechanisms, among which GSK3β appears to be a key player. Dephosphorylation of GSK3β leads to marked shrinkage of dendrites, whereas its inhibition (phosphorylation) enhances dendritic growth and GSK3β phosphorylation is required for proper synapse development (Jin et al., 2012, Kim and Snider, 2011, Rui et al., 2013). The finding that treatment with ELN increased GSK3β phosphorylation in Ts65Dn mice suggests that this effect may contribute to the restoration of neurite length, observed here in cultures on NPCs, and synapse development, observed in vivo.

### 4.3 Neonatal treatment with a γ-secretase inhibitor impacts APP processing in Ts65Dn mice

While a 24 h treatment with ELN did not change APP levels in trisomic NPCs (see Fig. 3C), in mice treated for 13 days there was a reduction in APP levels (see Fig. 8A, C). Since AICD promotes transcription of APP itself, the reduction of APP levels in mice treated with ELN may be attributable to a reduction in the AICD-mediated transcription of APP. It has been shown that inhibition of GSK3 decreases APP levels by enhancing its degradation via an increase in the number of lysosomes (Parr et al., 2012). In treated mice there was increased phosphorylation (i.e., increased inhibition) of GSK3β. Therefore, the reduction of APP levels in treated Ts65Dn mice may also be due to an increase in APP degradation. Since APP acts to markedly decrease NGF retrograde transport and cause degeneration of BFCNs (Salehi et al., 2006), the treatment induced reduction in APP levels may prevent cholinergic neurodegeneration.

### 4.4 Conclusions

Results show that inhibition of APP γ-secretase restores neurogenesis, hippocampal granule cell number and synaptic development in a mouse model of DS. We used a selective inhibitor of APP γ-secretase (ELND006) that was created in order to reduce Aβ formation in Alzheimer's disease (Basi et al., 2010, Probst et al., 2013). Our results suggest that inhibitors of APP γ-secretase may be exploited in order to improve neurogenesis defects in DS through a reduction in AICD formation. Indeed, early treatment with ELND006 restored neurogenesis in the two major neurogenic niches of the postnatal brain and development of the hippocampal dentate gyrus. ELND006 was developed as an APP selective γ-secretase inhibitor for the treatment of AD, and studies have shown that ELND006, at therapeutic doses, has no toxic effects on wild-type mice and nonhuman primates (Basi et al., 2010, Brigham et al., 2010), but a clinical trial with ELND006 in elderly healthy volunteers was discontinued due to elevation of liver enzyme levels, a sign of liver toxicity (Hopkins, 2011). Yet, though ELND006 by itself may not be a suitable drug for DS (or AD), our study provides novel demonstration that inhibitors of γ-secretase can completely reinstate neurogenesis in the trisomic brain. Intense research is being carried out in order to devise safe inhibitors of γ-secretase for the cure of Alzheimer's disease. For instance, a modulator of γ-secretase as well as direct inhibitor of AICD activity has recently been developed (Branca et al., 2014). All this evidence prospects that there will soon be the possibility to exploit safe drugs in order to reduce AICD levels/activity and correct the neurogenesis defects of DS.

## Conflict of interest

The authors declare that they have no conflict of interest.
